# Classification of bone flap resorption after cranioplasty: a proposal for a computed tomography-based scoring system

**DOI:** 10.1007/s00701-018-03791-3

**Published:** 2019-01-14

**Authors:** Tommi K. Korhonen, Niina Salokorpi, Pasi Ohtonen, Petri Lehenkari, Willy Serlo, Jaakko Niinimäki, Sami Tetri

**Affiliations:** 1 Department of Neurosurgery, Oulu University Hospital, Kajaanintie 52, 90029 Oulu, Finland; 2 Research Unit of Clinical Neuroscience, Neurosurgery, University of Oulu, Oulu, Finland; 3 Division of Operative Care, Oulu University Hospital, Oulu, Finland; 4 Department of Anatomy and Cell Biology and Department of Surgery, MRC Oulu, University of Oulu, Oulu University Hospital, Oulu, Finland; 5 PEDEGO Research Unit, MRC Oulu, University of Oulu, and Department of Children and Adolescents, Oulu University Hospital, Oulu, Finland; 6 Research Unit of Medical Imaging, Physics and Technology, University of Oulu, Oulu, Finland

**Keywords:** Cranioplasty, Cryopreservation, Decompressive craniectomy, Bone flap resorption, Computed tomography, Score

## Abstract

**Background:**

Bone flap resorption (BFR) is the most prevalent complication resulting in autologous cranioplasty failure, but no consensus on the definition of BFR or between the radiological signs and relevance of BFR has been established. We set out to develop an easy-to-use scoring system intended to standardize the interpretation of radiological BFR findings.

**Methods:**

All 45 autologous cranioplasty patients operated on at Oulu University Hospital from 2004 to 2014 were identified, and the bone flap status of all the available patients was evaluated using the new scoring system. Derived from previous literature, a three-variable score for the detection of BFR changes is proposed. The variables “Extent” (estimated remaining bone volume), “Severity” (possible perforations and their measured diameter), and “Focus” (the number of BFR foci within the flap) are scored from 0 to 3 individually. Using the sum of these scores, a score of 0–9 is assigned to describe the degree of BFR. Additionally, independent neurosurgeons assessed the presence and relevance of BFR from the same data set. These assessments were compared to the BFR scores in order to find a score limit for relevant BFR.

**Results:**

BFR was considered relevant by the neurosurgeons in 11 (26.8%) cases. The agreement on the relevance of BFR demonstrated substantial strength (*κ* 0.64, 95%CI 0.36 to 0.91). The minimum resorption score in cases of relevant BFR was 5. Thus, BFR with a resorption score ≥ 5 was defined relevant (grades II and III). With this definition, grade II or III BFR was found in 15 (36.6%) of our patients. No risk factors were found to predict relevant BFR.

**Conclusions:**

The score was proven to be easy to use and we recommend that only cases with grades II and III BFR undergo neurosurgical consultation. However, general applicability can only be claimed after validation in independent cohorts.

## Introduction

Due to the increase in decompressive craniectomy patient volumes in the recent decades [1, 2, 6, 18, 22], it is clear that increasing numbers of cranioplasty procedures are being performed. Having generally sustained a major neurological insult, cranioplasty patients are often extensively screened radiologically during their lifetime for disease relapses. Incidental BFR is a common finding in these scans [11], but the exact definition of relevant BFR is unclear [5, 8, 9].

In previous studies evaluating the radiological signs of bone flap resorption (BFR), varying stages of BFR have been reported from 43 to 53.6% of patients [4, 5, 21, 23], but not all of these were clinically relevant nor required reoperation. The retrospectively reported prevalence of clinically relevant BFR varies from 1.4% [10] to 32.0% [15]. This variation has been suggested to be due to unclarities in the definition and clinical relevance of radiological BFR findings [7, 9].

The varying severity of radiological BFR poses a challenge to the radiologists and the potentially non-neurosurgical clinicians, who determine whether the patient should be referred to a neurosurgical consultation. The clinical relevance of the radiologically diverse [23] evidence of BFR is currently unclear. This demands a robust definition of BFR and the establishment of the radiological signs of BFR that define the need to forward the patient to a neurosurgical unit.

In the present study, we propose a new computed tomography (CT)-based scoring system intended to standardize the evaluation of BFR between raters. We had two main goals: firstly, to precisely define relevant BFR, which has been a problem in previous studies [5, 7, 9], and secondly, to produce a scoring system that allows clinicians and radiologists to recognize clinically relevant BFR from CT data. A preliminary analysis using the scoring system was conducted on our cohort of patients that have undergone primary autologous cranioplasty after decompressive craniectomy. In the long term, adoption of the scoring system will decrease the number of unnecessary neurosurgical consultations and referrals due to BFR findings, which is favorable due to the concurrently increasing patient volumes.

## Methods and materials

### Development of the scoring system

The proposed score and grading schemes were designed collaboratively by the authors, with the ultimate goal of obtaining a robust and objective system that could be used to reliably define BFR, which has been described to be a diverse complication with various subtypes occurring even in the same bone flap [23]. Therefore, universal markers of BFR are required for the scoring system. Variables proposed to constitute the scoring system are “Extent” (estimated remaining bone volume), “Severity” (possible perforations and their measured diameter), and “Focus” (the number of BFR foci within the bone flap). The theoretical basis for the selection of these variables and their cutoff values is addressed in detail in the “Discussion” section of the present manuscript.

**Fig. 1:**
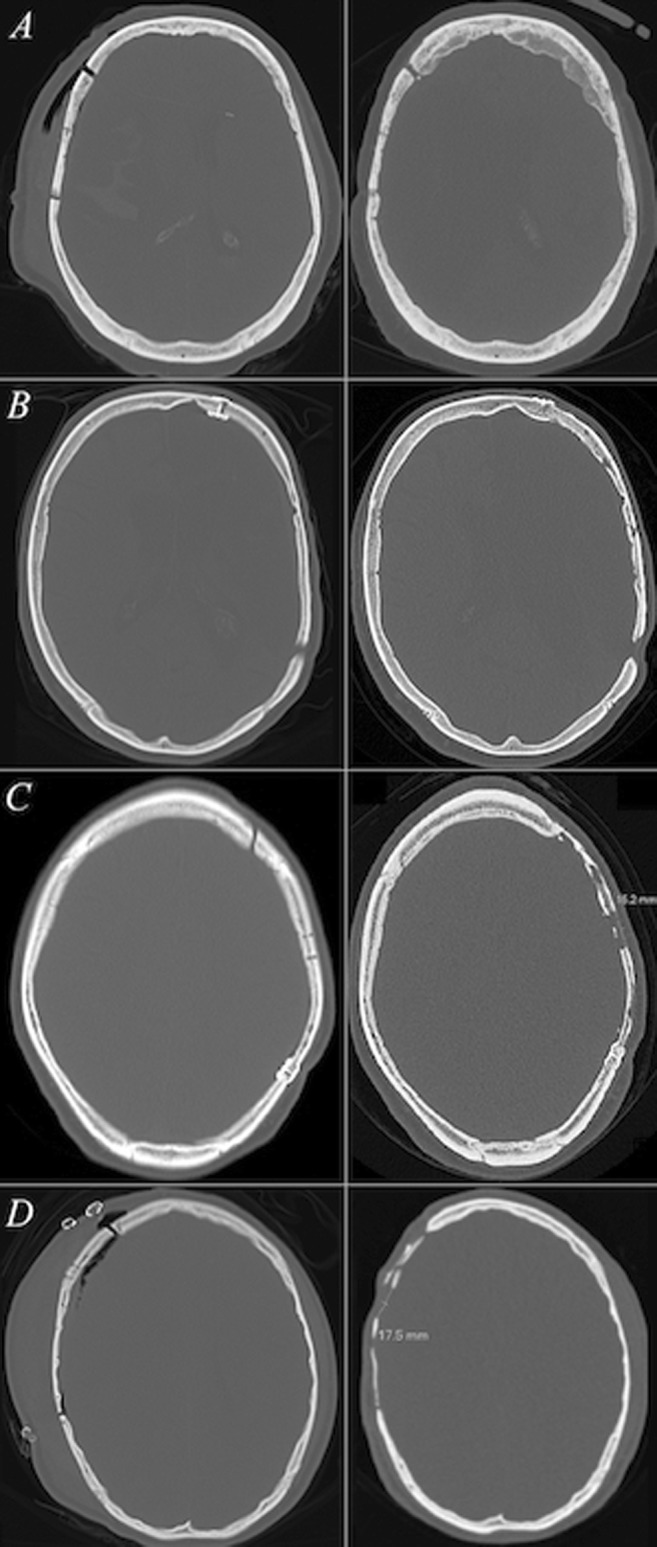
CT slices with bone window settings depict the initial postoperative and follow-up bone flap statuses (left and right columns, respectively) of four cranioplasty patients with different levels of bone flap resorption. Each row comprises one patient. In **a**, the follow-up bone flap status was given a mean Oulu resorption score of 0 (grade 0) as no signs of bone flap resorption were visible. The bone flap status of the patient in **b** was given a mean Oulu resorption score of 1.33 (grade I)—though the follow-up bone volume remained ≥ 75%, relatively mild bone flap resorption manifesting as multiple non-perforating cortical irregularities on both sides of the autograft was visible. More notable signs of bone flap resorption are depicted in **c**, where the follow-up status of the cranioplasty was graded a mean Oulu resorption score of 8 (grade II). The postoperative bone volume had decreased to 25–75% of the original volume along with diffuse resorptive changes on the bone flap area and a bicortical perforation exceeding 1 cm in diameter. Yet, the autograft had remained somewhat fixed to the cranium. In **d**, the cranioplasty had failed and the mean Oulu resorption score was 9 (grade III). The follow-up bone flap volume was < 25% of the original, bicortical perforations larger than 1.0 cm were noted and the resorptive changes within the bone flap were diffuse. Neither of the surgeons recommended further interventions for patients **a** or **b**. For the patient **c**, one surgeon recommended a neurosurgical consultation and for the patient **d**, both surgeons recommended consultation of a neurosurgeon and additionally a re-cranioplasty evaluation due to bone flap failure

Each variable was further divided into four subclasses and scored accordingly, and the resorption score was calculated as the sum of the subclass scores, with the minimum score being 0, and the maximum 9. Increasing values indicate more serious BFR (Fig. 1). A summary of the proposed three-variable scoring system is presented in Table 1.

**Table 1:** The proposed classification and scoring criteria for the Oulu resorption score as used in the analyses

Variable	Description	Score
Extent	Remaining bone volume	
	No BFR/remaining bone volume = 100.0%	0
	Remaining bone volume 75.0 to 99.9%	0
	Remaining bone volume 25.0 to 74.9%	2
	Remaining bone volume < 25.0%	3
Severity	Perforations due to BFR	
	No BFR or only cancellous bone loss	0
	Non-perforating resorption	1
	A new bicortical perforation of < 1.0 cm	2
	A new bicortical perforation of ≥ 1.0 cm	3
Focus	Total integrity of the bone flap	
	No BFR	0
	One focal BFR change	0
	Multiple BFR foci	2
	Diffuse BFR: signs of BFR throughout the flap area	3
Oulu resorption score: Σ=		

*BFR* bone flap resorption, *Σ* the sum of the Extent, Severity, and Focus scores, making up the Oulu resorption score

### Patient population

The new system was tested on our patient cohort. We identified all the patients on whom a decompressive craniectomy and subsequently a primary autologous cranioplasty had been performed at Oulu University Hospital, a tertiary-level teaching hospital, between 2004 and 2014 (*n* = 45). In order to ensure no data were missed, secondary centers were queried for additional CT scans. Patients were invited for a follow-up CT scan at Oulu University Hospital or a secondary center if their latest available CT scan was more than 1 year old. If the patient’s bone flap had already been removed, the pre-removal CT scan was used for the evaluation. Four patients (8.9%) had to be excluded, three (6.7%) due to death before the required CT scans could be taken, and one (2.2%) due to insufficient CT data, leaving a series of 41 patients (91.1% of total). A study on the current patient cohort has been published earlier [11].

### Radiological evaluation

Using the latest CT data for each patient, two of the authors (TKK and JN) independently evaluated and graded each patient’s bone flap status using the new scoring system. For comparison purposes, the raters were allowed access to the earliest post-cranioplasty head CT scan available. If no sufficient post-cranioplasty comparison scan was found, a pre-craniectomy CT scan was used for comparison. Prior to the classification process, the evaluators held a meeting to identify and resolve any discrepancies and ambiguities in the classification system. TKK re-scored the CT data in random order 1 month after the initial evaluation. The mean resorption scores were calculated based on these three evaluation rounds, and the mean scores were used for further statistical analyses.

Any classification system should confer value for clinical decision-making. In order to assess the viability of the scoring system, two neurosurgeons, each with at least 10 years of experience (ST and NS), independently assessed the same head CT data set to ascertain whether any signs of BFR were present, and whether the BFR was relevant, e.g., whether neurosurgical consultation or a re-cranioplasty evaluation should be recommended. A consultation was considered necessary if at least either of the evaluators recommended it.

The neurosurgeons’ evaluations on the relevance of the BFR findings were compared with the mean resorption scores of the patients in order to obtain preliminary threshold values for the scoring system. To produce a more user-friendly scoring system, the patients’ score results were divided into grades 0 (no BFR), I (non-relevant BFR), II (relevant BFR), and III (bone flap failure due to BFR).

### Radiological specifications

The CT data used for the analyses were evaluated using the hospital’s clinical radiology software (neaView Radiology, Neagen Ltd., Helsinki, Finland) with bone window settings (width 2800 and level 600 in Hounsfield Units). An approximate estimate of the bone flap size was obtained from the 2D scout image using the same software, which allowed for manual outlining of the bone flap and calculation of its area. All the CT data were stored in a picture archiving and communication system.

### Clinical data

The patients’ baseline characteristics were collected from the hospital records and consisted of sex, age at cranioplasty, dates of the craniectomy and cranioplasty procedures, complications (bone flap resorption, surgical site infections (SSI), hematoma/seroma, cerebrospinal fluid leak, poor cosmesis, hydrocephalus, implant migration, or exposure), and outcome. Additionally, data on smoking and primary diagnosis was collected.

### Statistical analysis

Summary baseline measurements are reported as mean with standard deviation (SD) and range or median with interquartile range (IQR). The neurosurgeons’ agreement on the presence and relevance of BFR was evaluated using Cohen’s kappa (*κ*), which is a statistical measure of observer agreement strength that also accounts for agreement by chance. The *κ* values are reported with 95% confidence intervals (CI). The values of *κ* were interpreted according to Landis and Koch [13], who classify values of < 0.0 as poor, 0.0–0.20 slight, 0.21–0.40 fair, 0.41–0.60 moderate, 0.61–0.80 substantial, and 0.81–1.00 almost perfect. Categorical variables were evaluated using the *χ*^2^ and Fisher’s exact tests with grade II BFR as the dependent variable. A two-tailed *p* value of < 0.05 was considered statistically significant. The analyses were performed using SPSS for Windows (IBM Corp. Released 2016. IBM SPSS Statistics for Windows, Version 24.0. Armonk, NY: IBM Corp).

### Ethical considerations

The study was performed in accordance with the declaration of Helsinki on ethical principles for medical research. The protocol was approved by the ethics review committee of the Northern Ostrobothnia hospital district, and patient consent was acquired for the follow-up CT scans. Patients whose follow-up CT scans that were used for the analyses showed alarming findings were contacted, examined clinically and counseled on the findings by the senior author.

## Results

### Descriptive data

Forty-one patients included in this study had undergone primary autologous cranioplasty after decompressive craniectomy. The baseline characteristics are reported in Table 2, and the postoperative complications in Table 3. The mean age at cranioplasty was 41 years (range 15–65 years, SD 14.8). The bone flaps underwent a mean freezer time of 207 days (range 1 to 538 days, SD 124). The CT images from which the BFR was evaluated were taken at a mean 4.3 years (SD 3.14, range 0.13–11.55 years) after the cranioplasty operation. The median 2D lateral bone flap area was 91.7 cm^2^ (IQR 34.8 cm^2^).

**Table 2:** The baseline characteristics of all the 41 patients with primary autologous cranioplasty after decompressive craniectomy and the number of patients with relevant bone flap resorption (grades II to III) defined as Oulu resorption score ≥ 5. Non-relevant BFR (grade 0 to I) is defined as Oulu resorption score < 5

Variable	Patients (*n* = 41)	Grades II to III BFR (*n* = 15)	Grades 0 to I BFR (*n* = 26)	*p* value
Sex				0.28
Male, *n* (%)	31 (75.6)	13 (86.7)	18 (69.2)	
Female, *n* (%)	10 (24.4)	2 (13.3)	8 (30.8)	
Age (years)				0.09
15–29	11 (26.8)	7 (46.7)	4 (15.4)	
30–49	16 (39.0)	5 (33.3)	11 (42.3)	
50–65	14 (34.1)	3 (20.0)	11 (42.3)	
Indication for decompressive craniectomy				0.60
Trauma	16 (39.0)	7 (46.7)	9 (34.6)	
Stroke	24 (58.5)	7 (46.7)	17 (65.4)	
Intracranial ischemia	19 (46.3)	6 (40.0)	13 (50.0)	
SAH	1 (2.4)	0 (0.0)	1 (3.8)	
ICH	4 (9.8)	1 (6.7)	3 (11.5)	
Other	1 (2.4)	1 (6.7)	0 (0.0)	
Smoking habit				0.15
Smoker, *n* (%)	14 (34.1)	3 (20.0)	11 (42.3)	
Non-smoker, *n* (%)	27 (65.9)	12 (80.0)	15 (57.7)	
2D defect size				0.13
< 91.70 cm^2^, *n* (%)	20 (48.8)	5 (33.3)	15 (57.7)	
≥ 91.70 cm^2^, *n* (%)	21 (51.2)	10 (66.6)	11 (42.3)	
Freezer time				0.46
< 90 days	8 (19.5)	4 (26.7)	4 (15.4)	
90 to 179 days	11 (26.8)	5 (33.3)	6 (23.1)	
≥ 180 days	22 (53.7)	6 (40.0)	16 (61.5)	

A *p* value of < 0.05 was considered statistically significant in the *χ*^2^ or Fisher’s exact tests

*BFR* bone flap resorption, *SAH* subarachnoid hemorrhage, *ICH* intracerebral hemorrhage

**Table 3:** Primary complications observed in 41 primary autologous cranioplasty operations

Clinical complication	Total number of patients (*n* = 41)	Bone flap removal required (*n* = 7)
Bone flap resorption, *n* (%)	4 (9.8)	4 (57.1)
Deep SSI, *n* (%)	3 (7.3)	3 (42.9)
Hematoma or seroma, *n* (%)	4 (9.8)	0 (0.0)
CSF leak, *n* (%)	5 (12.2)	0 (0.0)

*SSI* surgical site infection, *CSF* cerebrospinal fluid

### Evaluation of BFR using the scoring system

When evaluated using the proposed scoring system, nine (22.0%) of our patients had no signs of BFR, and thus their mean score was 0. A mean resorption score higher than zero, implying the presence of some degree of BFR, was found in 32 (78.0%) of the cases (Table 4).

**Table 4:** The mean Oulu resorption scores of 41 primary autologous cranioplasty patients as calculated from the independent radiological evaluations. Also described are the relevance of the radiological BFR changes evaluated by independent neurosurgeons, the grade of BFR derived from the score and the recommended action. Grade II or higher was defined as relevant BFR, and grade III BFR indicates bone flap failure. Grades 0 and I represent non-existent and non-relevant BFR, respectively

Mean Oulu resorption score	Non-relevant BFR (*n* = 30)*	Relevant BFR (*n* = 11)*	BFR grade	Action
0	9 (30.0)	0 (0.0)	0	None
0.1 to 0.9	3 (10.0)	0 (0.0)	I	None
1 to 1.9	5 (16.7)	0 (0.0)		
2 to 2.9	2 (6.7)	0 (0.0)		
3 to 3.9	5 (16.7)	0 (0.0)		
4 to 4.9	2 (6.7)	0 (0.0)		
5 to 5.9	4 (13.3)	2 (18.2)	II	Consult
6 to 6.9	0 (0.0)	3 (27.3)		
7 to 7.9	0 (0.0)	1 (9.1)		
8 to 8.9	0 (0.0)	3 (27.3)		
9	0 (0.0)	2 (18.2)	III	Consult (bone flap failure)

*Data given as *n* (%)

*BFR* bone flap resorption

### Agreement on the presence and relevance of BFR

The neurosurgeons’ assessments pointed to some degree of BFR in the bone flaps in 31 (75.6%) and 34 (82.9%) cases. BFR was considered relevant by both neurosurgeons in 6 cases (14.6%) and by only one surgeon in 5 (12.2%). Thus it may be said that in total, relevant BFR was found by the neurosurgeons in 11 out of the 41 cases (26.8%).

The surgeons’ agreement on the presence of any level of BFR and the relevance of BFR is reported in Tables 5 and 6. The agreement demonstrated substantial strength concerning both the presence and relevance of BFR (respective *κ* values of 0.63 (95% CI 0.34 to 0.92) and 0.64 (95% CI 0.36 to 0.91)).

**Table 5:** Presence of any level of bone flap resorption as assessed by two independent neurosurgeons

	BFR present, evaluator 2	No BFR, evaluator 2
BFR present, evaluator 1	30	4
No BFR, evaluator 1	1	6

*BFR* bone flap resorption

**Table 6:** The agreement on the relevance of the BFR findings as independently evaluated by two neurosurgeons

	Relevant BFR, evaluator 2	Non-relevant BFR at most, evaluator 2
Relevant BFR, evaluator 1	6	0
Non-relevant BFR at most, evaluator 1	5	30

*BFR* bone flap resorption

### The grade of BFR

All of the 11 patients whose BFR status was considered relevant by the neurosurgeons had a mean resorption score ≥ 5 (Table 4). Additionally, 4 patients with a mean resorption score ≥ 5 were evaluated by the neurosurgeons as having non-relevant BFR. In order for the system to identify all patients with relevant BFR, we propose a resorption score of ≥ 5 as the threshold value for relevant BFR (grades II and III). Thus, 15 patients (36.6%) had relevant BFR, which indicated radiological BFR severe enough to likely demand clinical interventions as evaluated by the neurosurgeons. Both of the patients for whom the neurosurgeons recommended to consider re-cranioplasty evaluation due to bone flap failure had reached a mean resorption score of 9, which is proposed as the threshold value of grade III BFR (Table 4).

### Clinical data and relevant BFR

The evaluation of risk factors for relevant BFR (resorption score ≥ 5) is depicted in Table 2. No statistically significant associations between relevant BFR and sex, age, primary diagnosis, smoking habits, 2D defect size, or the duration of freezer time between craniectomy and cranioplasty were observed.

## Discussion

Due to the recent increase in decompressive craniectomy patient volumes [1, 2, 6, 18, 22], cranioplasty operations are increasing in numbers. Having generally sustained a severe neurosurgical condition, cranioplasty patients undergo extensive radiological follow-up for disease recurrences. Thus, incidental BFR findings such as those depicted in Fig. 1 are increasingly emerging in CT scans as time passes. A recent study revealed some degree of BFR in up to 90% of cases [11], supporting the early findings of Prolo et al. [16].

Without a straight-forward scoring system, it can be rather challenging for the clinician or radiologist to identify the cases that should be referred to a neurosurgeon for evaluation, especially as by the time BFR can be detected, the patients are being treated in local hospitals and rehabilitation facilities with limited neurosurgical ability. A robust definition of BFR is required for reliable analysis of risk factors for BFR between future studies. The Oulu resorption score was developed to standardize the interpretation of incidental BFR for both research and clinical purposes.

### Theoretical basis of the scoring system

The variables proposed to constitute the Oulu resorption score system (Table 1) are based on the three previously published attempts to classify radiological BFR findings [4, 21, 23] and a recent volumetric study [11].

All of the previous CT-based attempts to classify BFR consider thinning of the bone as a marker of BFR [4, 21, 24]. In a previous study [11], quantitative bone flap volumes of less than 32.2% of the original volume manifested as aseptic BFR severe enough to indicate removal of the bone flap. Based on this, the lower cutoff value for the estimated remaining bone volume, the “Extent” variable, was chosen to be 25.0%. In the same study, 73.2% of the patients had a bone flap volume of ≥ 75.0%, which is suggested to be the upper cutoff value for the estimated bone volume variable.

In addition to the loss of bone volume, perforations in the bone flap deteriorate both the functional [8] and cosmetic outcomes of cranioplasty and are accounted for in the score using the “Severity” variable. A perforation of the size of a burr hole may already produce cosmetic issues [3]. Thus, a diameter of 1.0 cm, which has been used as a marker of BFR in a previous study [9], is proposed as the cutoff value for the “Severity” variable. As bone flaps may contain burr holes or other iatrogenic defects, we suggest that only new perforations or enlargement of the existing ones are taken as markers of BFR.

The total portion of the bone flap affected by the resorption process is also reflected in two of the previous classification systems [4, 23], but a more objective description of the stage of BFR would probably better correlate with the total integrity of the bone flap. Therefore, we propose that the number of BFR foci within the bone flap is accounted for in the scoring system by way of the “Focus” variable, as one focal resorption change is clinically less significant than a diffusely resorbed bone flap. As a moderate degree of BFR is associated with the revitalization process of the bone flap after cranioplasty [16], a remaining bone volume of over 75.0% and the presence of at most one focal BFR change were both interpreted physiological and therefore scored 0 points.

The cutoff value of ≥ 5 for relevant BFR was obtained by comparing the patients’ mean resorption scores with the independent evaluations on the relevance of BFR conducted by two neurosurgeons. To ensure the viability of this threshold, the agreement of the neurosurgeons was tested with the *κ* statistic. The agreement demonstrated substantial strength with a *κ* value of 0.64. Additionally, the neurosurgeons recommended re-cranioplasty evaluation based on the CT images for two patients with a resorption score of 9. This was defined as the cutoff value for grade III BFR, which represents bone flap failure.

### The prevalence and risk factors of BFR

When the CT images of our autologous cranioplasty patients were evaluated independently using this scoring system, some degree of BFR (grades I–III) was found in 32 out of 41 cases (78.0%). Correspondingly, the two neurosurgeons independently noted any level BFR in 79.3% of cases on average. Radiological BFR with an Oulu resorption score of ≥ 5 was defined relevant, and it was found in 36.6% of our patients, which suggests that the Oulu resorption score successfully ruled out mild cases of BFR from the relevant BFR group. Though more robustly defined, the prevalence of BFR using the Oulu resorption score is in line with previous works assessing the radiological manifestations of BFR [4, 5, 8, 9, 20, 21, 23].

Based on our findings, we recommend that patients with grade II or III BFR should be referred to a neurosurgeon for consultation. Further, grade III BFR indicates failure of the bone flap, and replacement of the autologous cranioplasty with a synthetic implant should be considered by the neurosurgeon in at least these cases.

The proportion of patients with grade II or III BFR (Oulu resorption score ≥ 5) seemed to decrease with increasing patient age (Table 2), but this result did not reach statistical significance due to the small size of the age groups. Smoking was not associated with an increased prevalence of grade II or III BFR, but an earlier report demonstrated smoking to have a detrimental effect for autologous cranioplasty outcome mainly through increased SSI rates [12]. Additionally, no associations between relevant BFR and sex, primary diagnosis, freezer time (< 90, 90–180, > 180 days), or 2D craniectomy area (over or under the median 91.7 cm^2^) were found. Of these, young age is a commonly accepted predictor of BFR, and the results reported earlier on the other factors are thus far inconclusive [5, 12, 14, 17, 19].

### Implications of the Oulu resorption score

The reliability and clinical applicability of the present scoring system would be an interesting topic for future research, since adoption of the proposed resorption score should offer possibilities for limiting the number of CT follow-ups and avoiding unjustified neurosurgical consultations and referrals arising from unclarity in the interpretation of BFR findings, which is an important consideration with the increasing patient volumes. Additionally, a standardized BFR classification system facilitates accurate identification of risk factors of BFR and enhances inter-study comparison thus ultimately improving future research quality.

As the essence of the process of developing radiological grading systems and treatment protocols lies in the fact that it is an iterative process, future comments and suggestions for modifications to the presently proposed scoring system will be important and are to be welcomed. Further, the scoring system requires validation in terms of reliability using independent patient cohorts. The validation of the present scoring system is the subject of a subsequent study, and we are looking to welcome additional centers for a multicenter validation study of the scoring system.

### Strengths and weaknesses

The strength of this work is that it accurately represents our autologous cranioplasty patient cohort from 2004 to 2014, as it includes 91.1% of the patients operated on during that period in the Oulu University Hospital. The proposed system for BFR scoring requires a minimum of subjective decisions, and it is based on the relevant previously published classification and bone flap volumetry studies.

The clinical data was collected retrospectively in the present study, and thus the inherent weaknesses of retrospective study design apply. The present study population, although accurately represented, is limited in size and thus the effect of chance may be prominent in both the prevalence of BFR and observer agreement. Additionally, the length of follow-up varied between patients and consequently the time of recording the Oulu resorption score was not constant, which may have influenced the results. Nevertheless, differences were recognized by the scoring system. The youngest patient who underwent autologous cranioplasty after decompressive craniectomy was 15 years old. Thus, we could not extend our results to pediatric patients younger than 15 years of age. The cutoff age for emergency decompressive craniectomy in our institution is 65 years, and the study cohort did not contain patients over that age. Further, patient-dependent variables, especially age may have influenced the size of the bone defect and other variables measured in the present study. Despite being a good tool for evaluating CT images, the present system only accounts for BFR, and this score alone does not suffice for determining whether a re-operation is necessary, but a clinical evaluation is also required.

## Conclusions

The Oulu resorption score aims to standardize the interpretation of post-cranioplasty BFR in CT scans. Cases of BFR considered relevant by independent neurosurgeons were successfully recognized by the scoring system. We recommend that only grades II and III BFR cases undergo neurosurgical consultation. Ultimately, the general applicability of the Oulu resorption score will depend on validation employing independent patient cohorts.

## Abbreviations

BFR: Bone flap resorption
CI: Confidence interval
CT: Computed tomography
IQR: Interquartile range
*κ*: Cohen’s kappa
SD: Standard deviation
SSI: Surgical site infection

## References

[1] Adeoye O, Hornung R, Khatri P, Ringer A, Kleindorfer D (2011) The rate of hemicraniectomy for acute ischemic stroke is increasing in the United States. J Stroke Cerebrovasc Dis. 10.1016/j.jstrokecerebrovasdis.2010.01.006 10.1016/j.jstrokecerebrovasdis.2010.01.006 20621514

[2] Bhattacharya P, Kansara A, Chaturvedi S, Coplin W (2013) What drives the increasing utilisation of hemicraniectomy in acute ischaemic stroke? J Neurol Neurosurg Psychiatry. 10.1136/jnnp-2012-303610 10.1136/jnnp-2012-303610 23412075

[3] Dujovny M, Aviles A, Agner C, Fernandez P, Charbel FT (1997). Cranioplasty: cosmetic or therapeutic?. Surg Neurol.

[4] Dünisch P, Walter J, Sakr Y, Kalff R, Waschke A, Ewald C (2013). Risk factors of aseptic bone resorption: a study after autologous bone flap reinsertion due to decompressive craniotomy. J Neurosurg.

[5] Ernst G, Qeadan F, Carlson AP (2018) Subcutaneous bone flap storage after emergency craniectomy: cost-effectiveness and rate of resorption. J Neurosurg 129:1604–1610 10.3171/2017.6.JNS17943 29303450

[6] Honeybul S, Ho KM (2013). The current role of decompressive craniectomy in the management of neurological emergencies. Brain Inj.

[7] Honeybul S, Ho KM (2016) Cranioplasty: morbidity and failure. Br J Neurosurg. 10.1080/02688697.2016.1187259 10.1080/02688697.2016.1187259 27215939

[8] Honeybul S, Morrison DA, Ho KM, Lind CRP, Geelhoed E (2017) A randomized controlled trial comparing autologous cranioplasty with custom-made titanium cranioplasty. J Neurosurg. 10.3171/2015.12.JNS152004 10.3171/2015.12.JNS152004 26991387

[9] Kim JH, Kim JH, Kwon T, Chong K, Hwang S, Yoon WK (2018) Aseptic bone flap resorption after cranioplasty with autologous bone: incidence, risk factors, and clinical implications. World Neurosurg. 10.1016/j.wneu.2018.03.197 10.1016/j.wneu.2018.03.197 29626687

[10] Klinger DR, Madden C, Beshay J, White J, Gambrell K, Rickert K (2014) Autologous and acrylic cranioplasty: a review of 10 years and 258 cases. World Neurosurg. 10.1016/j.wneu.2013.08.005 10.1016/j.wneu.2013.08.005 24036124

[11] Korhonen TK, Salokorpi N, Niinimäki J, Serlo W, Lehenkari P, Tetri S (2018a) Quantitative and qualitative analysis of bone flap resorption in patients undergoing cranioplasty after decompressive craniectomy. J Neurosurg. 10.3171/2017.8.JNS171857 10.3171/2017.8.JNS171857 29473777

[12] Korhonen TK, Tetri S, Huttunen J, Lindgren A, Piitulainen JM, Serlo W, Vallittu PK, Posti JP (2018b) Predictors of primary autograft cranioplasty survival and resorption after craniectomy. J Neurosurg. 10.3171/2017.12.JNS172013 10.3171/2017.12.JNS172013 29749908

[13] Landis JR, Koch GG (1977) The measurement of observer agreement for categorical data. Biometrics 33:159–174 843571

[14] Malcolm JG, Rindler RS, Chu JK, Grossberg JA, Pradilla G, Ahmad FU (2016) Complications following cranioplasty and relationship to timing: a systematic review and meta-analysis. J Clin Neurosci. 10.1016/j.jocn.2016.04.017 10.1016/j.jocn.2016.04.017 27499122

[15] Moreira-Gonzalez A, Jackson IT, Miyawaki T, Barakat K, DiNick V (2003) Clinical outcome in cranioplasty: critical review in long-term follow-up. J Craniofac Surg 14:144–153 10.1097/00001665-200303000-00003 12621283

[16] Prolo DJ, Burres KP, McLaughlin WT, Christensen AH (1979) Autogenous skull cranioplasty: fresh and preserved (frozen), with consideration of the cellular response. Neurosurgery 4:18–29 10.1227/00006123-197901000-00005 450211

[17] Rocque BG, Amancherla K, Lew SM, Lam S (2013) Outcomes of cranioplasty following decompressive craniectomy in the pediatric population. J Neurosurg Pediatr. 10.3171/2013.4.PEDS12605 10.3171/2013.4.PEDS12605 23790219

[18] Sahuquillo J, Arikan F (2006) Decompressive craniectomy for the treatment of refractory high intracranial pressure in traumatic brain injury. Cochrane Database Syst Rev. 10.1002/14651858.CD003983.pub2 10.1002/14651858.CD003983.pub2 16437469

[19] Schuss P, Vatter H, Oszvald A, Marquardt G, Imöhl L, Seifert V, Güresir E (2013) Bone flap resorption: risk factors for the development of a long-term complication following cranioplasty after decompressive craniectomy. J Neurotrauma. 10.1089/neu.2012.2542 10.1089/neu.2012.2542 22970998

[20] Schwarz F, Dünisch P, Walter J, Sakr Y, Kalff R, Ewald C (2016) Cranioplasty after decompressive craniectomy: is there a rationale for an initial artificial bone-substitute implant? A single-center experience after 631 procedures. J Neurosurg. 10.3171/2015.4.JNS159 10.3171/2015.4.JNS159 26406796

[21] Stieglitz LH, Fung C, Murek M, Fichtner J, Raabe A, Beck J (2015) What happens to the bone flap? Long-term outcome after reimplantation of cryoconserved bone flaps in a consecutive series of 92 patients. Acta Neurochir. 10.1007/s00701-014-2310-7 10.1007/s00701-014-2310-7 25534126

[22] Winter CD, Adamides A, Rosenfeld JV (2005) The role of decompressive craniectomy in the management of traumatic brain injury: a critical review. J Clin Neurosci. 10.1016/j.jocn.2005.02.002 10.1016/j.jocn.2005.02.002 16033709

[23] Zhang J, Peng F, Liu Z, Luan J, Liu X, Fei C, Heng X (2017a) Cranioplasty with autogenous bone flaps cryopreserved in povidone iodine: a long-term follow-up study. J Neurosurg. 10.3171/2016.8.JNS16204 10.3171/2016.8.JNS16204 28186447

[24] Zhang D, Xue Q, Chen J, Dong Y, Hou L, Jiang Y, Wang J (2017b) Decompressive craniectomy in the management of intracranial hypertension after traumatic brain injury: a systematic review and meta-analysis. Sci Rep. 10.1038/s41598-017-08959-y 10.1038/s41598-017-08959-y PMC5562822 28821777

